# *Lactobacillus Brevis* OPK-3 from Kimchi Prevents Obesity and Modulates the Expression of Adipogenic and Pro-Inflammatory Genes in Adipose Tissue of Diet-Induced Obese Mice

**DOI:** 10.3390/nu12030604

**Published:** 2020-02-26

**Authors:** Jung Eun Park, Suk-Heung Oh, Youn-Soo Cha

**Affiliations:** 1 Department of Food Science and Human Nutrition, Jeonbuk National University and Obesity Research Center, Jeonju, 54896 Jeonbuk, Korea; jpark3@jbnu.ac.kr; 2 Department of Food and Biotechnology, Woosuk University, Samnye-eup, Wanju-gun 55338, Jeonbuk, Korea; shoh@woosuk.ac.kr

**Keywords:** kimchi, *L. brevis* OPK-3, Obesity, inflammatory, C57BL/6N mice

## Abstract

Our previous study reported that lactic acid bacteria (*L. brevis* OPK-3) isolated from kimchi ameliorated intracellular lipid accumulation in 3T3-L1 adipocyte. The current study explored potential roles of *L. brevis* OPK-3 (KLAB) on preventing body weight gain and its effect on the inflammatory response of adipose tissue. Male C57BL/6 mice (*n* = 10) were divided into four groups: normal diet with distilled water (NDC), high-fat diet with distilled water (HDC), high-fat diet with L-ornithine (OTC) or high-fat diet with KLAB. The KLAB supplement resulted in significantly lower body weight, lower epididymal fat tissue mass, and lower serum and hepatic TG levels than the HDC. KLAB supplementation improved serum cytokines, and real-time polymerase chain reaction (PCR) analysis showed significantly lower inflammatory cytokine mRNA levels in epididymal adipose tissue. These results suggest that the administration of KLAB inhibits the induction of inflammation in adipose tissue along with the inhibition of weight gain. Therefore, this study demonstrates the therapeutic and beneficial value of this strain produced during the fermentation of kimchi.

## 1. Introduction

Obesity has increased dramatically with the economic development of society over the past few decades, and it has become one of the major health problems in the world [1]. It is associated with increased development of hyperinsulinemia, hyperglycemia, dyslipidemia, and non-alcoholic fatty liver disease (NAFLD) [2,3]. Low-grade chronic inflammatory response is responsible for the dysfunction of white adipose tissue (WAT). This, over time, leads to systemic inflammation. WAT releases adipokines, including inflammatory cytokines that regulate appetite, satiety, glucose metabolism, and inflammatory reactions. Cellular signaling pathways that are involved between adipokines and cytokines secreted from WAT have been reported to be altered by obesity, contributing to obesity-associated inflammation metabolism [4,5]. Adipose tissue inflammation in obesity is characterized by an increase in pro-inflammatory cytokines, such as tumor necrosis factor-α (TNFα), interleukin-6 (IL-6), and monocyte chemotactic protein-1 (MCP-1), along with macrophage or T immune cell accumulation, which contribute to the development and exacerbation of type 2 diabetes mellitus (T2DM), hypertension, atherosclerosis, and other metabolic disorders [6,7]. Therefore, the regulation of adipose tissue inflammation is significant to improve the obesity-associated metabolic disease.

Kimchi is a Korean traditional fermented vegetable and is a popular side dish that is consumed worldwide [8]. The taste of kimchi depends on the ingredients and the microorganisms involved in the fermentation process, along with the fermentation condition [9]. Kimchi is usually aged by natural fermentation at low temperatures. During this process, various lactic acid bacteria (LAB) increase with the onset of acid production, such as *Weissella koreensis*, *Leuconostoc kimchii*, *Lactobacillus brevis*, *Lactococcus lactis*, *Lactobacillus curvatus*, *Lactobacillus plantarum*, *Lactobacillus sakei*, *and Weissella kimchi* [10,11,12].

Among these lactic acid bacteria, in particular, strains of the genus *Lactobacillus* have been commercialized as probiotics in recognition of their great potential. *Lactobacilli* and *bifidobacteria* are well-known probiotics that have been reported to positively regulate immune responses [13], prevent cancer [14], improve intestinal functions [15] and have expert hypocholesterolemic effects [16]. Recent studies in various cellular and animal models have shown that even inactive probiotic cells could provide obvious health benefits to host animals [17]. Some studies have demonstrated the anti-obesity properties of probiotic bacteria by reducing the adipocyte size and regulating lipid and glucose metabolism [18,19,20,21]. In addition, during the fermentation and storage of kimchi, the active growth of LAB produces clinically important amino acids such as aminobutyric acid (GABA) and ornithine [22,23]. Among them, *Lactobacillus brevis*, a functional probiotic strain, has unique biochemical characteristics, which are closely associated with arginine deaminase activity. In particular, many LAB that produce ornithine, a metabolite of arginine, have been isolated from kimchi [24,25]. Ornithine affects the production of growth hormones, which promotes the metabolism of nutrients, such as carbohydrates, proteins, and lipids [26]. *Weissella koreensis* OK1-6 isolated from kimchi produced ornithine, which inhibited the accumulation of intracellular lipids during adipocyte differentiation [25]. We observed that *L. brevis* OPK-3 used in this study inhibits lipogenic and adipogenic genes in 3T3-L1 adipocyte [27] based on observation. These results suggest that lactic acid bacteria may improve adipose tissue inflammation in obesity. The present study was conducted to evaluate the improvement ornithine-producing *L. brevis* OPK-3 has on obesity and the inflammatory response isolated from kimchi on mice fed a high-fat diet. We hypothesized that KLAB supplementation could suppress high fat diet-induced obesity by modulating the expression profiles of lipid metabolism-related genes in the liver and white adipose tissue.

## 2. Materials and Methods

### 2.1. Preparation of Bacterial Cultures

*L*. *brevis* OPK-3 isolated from kimchi (KLAB) was offered from Genetic Engineering Laboratory, Woosuk University. The isolated KLAB was incubated in MRS broth (Difco, Detroit, MI, USA) containing arginine ((4%, v/v) ± 1% (w/v)). The strains were collected twice with neutral saline, and all mice received 1 × 10^9^ CFU/mL of the KLAB by oral gavages. Previous studies have shown that *Lactobacillus* was clinically safe when administered 10^7^ to 10^11^ CFU per day and was not associated with any intolerance problem [28]. In this study, the dosage of KLAB used was based on previous studies, clinical trials, or animal studies with *Lactobacillus* [29,30,31].

### 2.2. Animals and Diets

Four weeks old male C57BL/6N mice was bought from the Charles River Laboratories (Tokyo, Japan). After adaptation for a week, five-week-old mice were randomly divided into four groups: normal diet (NDC), high-fat diet (HDC), high-fat diet plus L-ornithine (OTC, 20 mg/kg BW per day), or high-fat diet plus *L*. *brevis* OPK-3 (KLAB, 1 × 10^9^ CFU per day). The diet composition is shown in Table 1. L-ornithine was used as a positive control. In this study, the L-ornithine dosage used was based on previous clinical trials and animal studies using L-ornithine. Each group was administered L-ornithine or *L*. *brevis* OPK-3 by oral gavage, while NDC or HDC received an equivalent volume of distilled water (DW) daily for 12 weeks. The mice were housed in 12 h light/dark cycle and a temperature controlled environment. After 12 weeks, blood and tissue samples were collected after anesthesia. The experimental protocol was approved by the Institutional Animal Care and Use Committee of Jeonbuk National University (CBNU 2011-0057).

### 2.3. Oral Glucose Tolerance Test (OGTT)

All mice were orally administrated 1g/kg of glucose after 12 h of overnight fasting. Blood samples were collected from the tail vein at baseline (0), 30, 60, and 120 min post-administration. The areas under the glucose curve (AUC) was calculated by the trapezoidal rule. Blood glucose levels were measured using a glucometer (Roche Diagnostics GmbH, Mannheim, Germany).

### 2.4. Lipid Profiles Assay and Hepatic Histology

Total cholesterol (TC) and triglyceride (TG) were measured using a commercial ELISA kit (Asan Pharmaceutical Co., Seoul, Korea). The HDL-cholesterol (HDL-C) fraction was measured using the dextran sulfate-Mg^++^ method. Hepatic lipid was extracted according to Folch’s method [32]. For histology, collected liver from mice was fixed in a 10% formalin solution for 48 h, and then embedded in paraffin. The prepared paraffin blocks were sectioned and stained with hematoxylin and eosin (H&E).

### 2.5. Detection of Cytokine Production

The serum concentrations of TNFα, IL-6, and IL-1β were determined with commercial ELISA kits (R&D Systems, Minneapolis, MN, USA). 

### 2.6. RNA Isolation and Hybridization of Microarray

Total RNA was extracted from the liver using the Trizol reagent (Invitrogen, Carlsbad, CA, USA). Quantification of total RNA was detected using NanoDrop ND-1000 (Nano-Drop Technologies, Wilmington, DE, USA). For microarray analysis, fluorescent-labeled cDNA was prepared by reverse transcription of total RNA in the presence of the Cy3-dUTP or Cy5-dUTP coupled (NEN) using Superscript II (Invitrogen, Carlsbad, CA, USA). Purified single-stranded cDNA probes were resuspended in hybridization buffer containing 50% formamide, 5X SSC, and 0.1% SDS. The 10K Mouse oligo chip (GenomicTree, Daejeon, Korea) was hybridized with the fluorescent-labeled.

### 2.7. Analysis of Microarray Data 

The hybridization images were captured with GenePix 4000B (Axon Instruments, Terumo, CA, USA) and digitized by GenePix Pro 6.0 program (Axon Instruments). The average fluorescence intensity of each spot was calculated, and the local background was subtracted. All data normalization and the selection of the genes showing fold changes were performed with GeneSpring GX 7.3.1 (Silicon Genetics, Redwood City, CA, USA). The identified genes were filtered with a cut-off value based on the two-component error model after intensity-dependent normalization (LOWESS). The averages of the normalized ratio were calculated as the ratio of mean normalized signal channel intensity to mean normalized control channel intensity.

### 2.8. Quantitative Real-Time PCR 

Total RNA was reversely transcribed into cDNA with a cDNA synthesis kit (Applied Biosystems, Foster City, CA, USA). Real-time PCR was performed in the 7500 Real-Time PCR system (Applied Biosystems, Foster City, CA, USA) using SYBR Green PCR Master Mix (Applied Biosystems, Woolston, Warrington, UK). The relative gene expression was calculated by normalization with β-actin used as an internal control. The primer sequences used in this were obtained from PrimerBank (http://pga.mgh.harvard.edu/primerbank). 

### 2.9. Statistical Analysis 

The data are expressed as means ± SD. One-way analysis of variance (ANOVA) was used for statistical comparisons with SPSS 17.0. The differences among the groups were determined using Duncan’s multiple range tests. All differences were considered significant with *p* < 0.05.

## 3. Results

### 3.1. Changes in Body Weight and Tissue Weight

**Figure 1:**
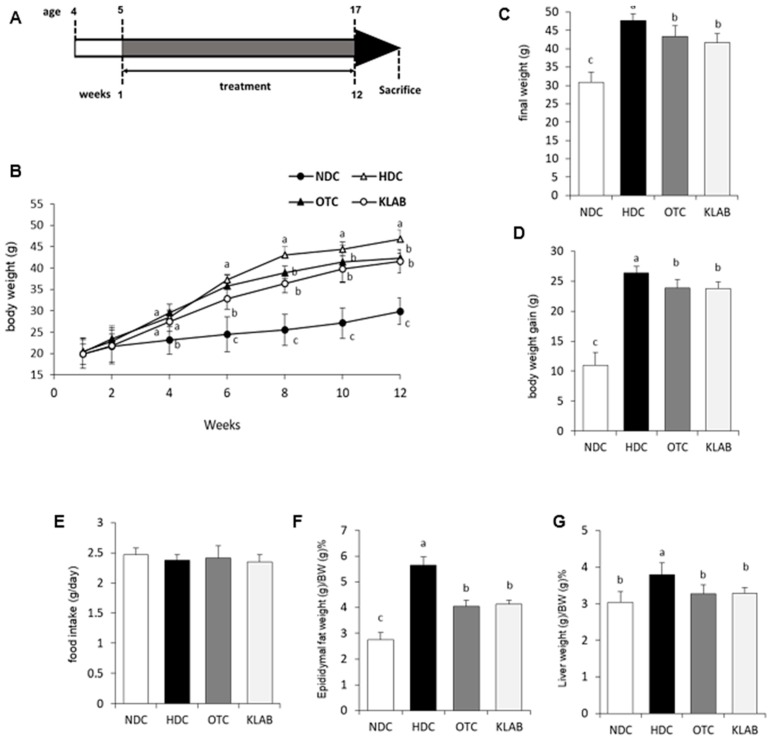
Scheme of experimental protocol, body weight and body composition of mice fed the experimental diet. Scheme of experimental protocol (**A**); growth curve (**B**); final weight (**C**); body weight gain (**D**); food intake (**E**); Epididymal fat weight (g)/BW (g) % (**F**); Liver weight (g)/BW (g) % (**G**). Results are Mean ± S.D of 10 mice per group. a-b-c; Values with different superscripts are significantly different by ANOVA with Duncan’s multiple range test at *p* < 0.05. NDC: Normal diet control; HDC: High fat diet control; OTC: high-fat diet plus L-ornithine 20 mg/kg; KLAB: High fat diet plus 1 × 10^9^ CFU of *L. brevis* OPK-3 per mouse. one-way ANOVA, analysis of variance.

The weight gain and food intake are shown in Figure 1. Body weight significantly increased in HFD-fed mice compared with mice fed a normal diet (*p* < 0.05) (Figure 1B,C). Feeding mice with HDC for 12 weeks increased body weight gain (40% increase) (Figure 1D), and relative epididymal adipose tissue and liver weight, compared with the mice fed a normal diet (Figure 1F,G). However, among the HFD-fed groups, the OTC and KLAB groups gained significantly less body weight, which was 10% less than in the HDC group. There was no difference in food intake. The epididymal adipose tissue and liver weights were significantly decreased in the OTC (35 and 17% of HDC) and KLAB (36 and 10% of HDC) groups compared with the HDC group (*p* < 0.05). These data suggest that KLAB intake could reduce adiposity in mice fed a high-fat diet. 

### 3.2. Blood Glucose Measurements

**Figure 2:**
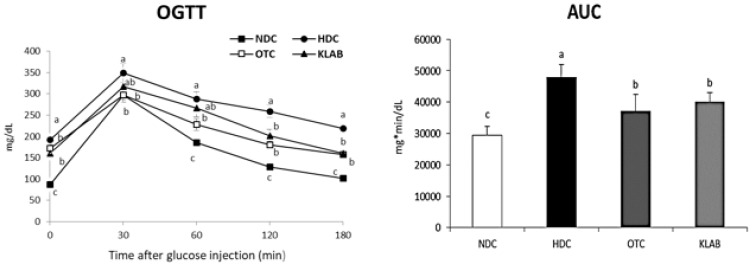
Oral glucose tolerance test (OGTT) and area under the curve (AUC). a-b-c; Values with different superscripts are significantly different by ANOVA with Duncan’s multiple range test at *p* < 0.05. NDC: Normal diet control; HDC: High fat diet control; OTC: high-fat diet plus L-ornithine 20 mg/kg; KLAB: High fat diet plus 1 × 10^9^ CFU of *L. brevis* OPK-3 per mouse.

We tested glucose tolerance with OGTT to evaluate the glycemic control effect of KLAB. (Figure 2). HFD-fed mice exhibited significantly higher glucose responses during the glucose tolerance test than in NDC mice (*p* < 0.05). However, OGTT and area under the curve (AUC) indicated an improved state of glucose tolerance in both the OTC and KLAB groups compared with the HDC group (Figure 2).

### 3.3. Serum and Hepatic Lipids

Lipid profiles in serum and liver are shown in Table 2. Mice fed a high-fat diet showed significantly higher levels of serum TG, TC, and hepatic TG content than in the NDC (*p* < 0.05). The levels of serum TG and TC were significantly decreased in the OTC and KLAB than in the HDC. Serum HDL-cholesterol (HDL-C) in the OTC and KLAB were significantly higher than in the HDC, and the HDL-C to TC ratios were not significant differences among the groups. Hepatic TGs were 38% lower in mice fed the KLAB than in those in HDC (*p* < 0.001). The positive control OTC showed significantly higher hepatic TGs level than in the KLAB group. 

### 3.4. Histopathology of Liver

**Figure 3:**
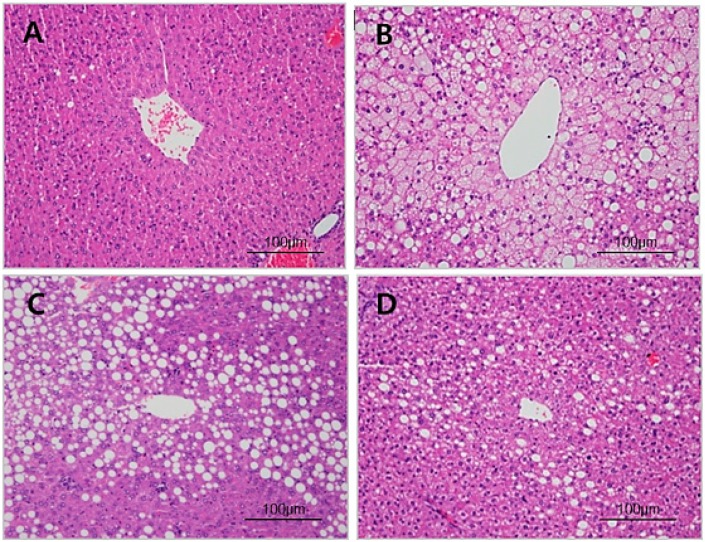
Histology of liver section. Histological examination (×100) in rat liver of (**A**) NDC (normal diet control group), (**B**) HDC (high-fat diet control group), (**C**) ORC (high-fat diet plus L-ornithine 20 mg/kg), and (**D**) KLAB (high-fat diet plus 1 × 10^9^ CFU of *L. brevis* OPK-3).

Histological examination confirmed significant lipid accumulation in the liver sections from mice of the HDC. Consistent with the reduced hepatic TG in mice, the lipid droplet infiltration in the liver section of the KLAB group was lower than in the HDC and OTC groups. (Figure 3), suggesting that constant KLAB supplementation protects HFD-induced lipid accumulation in the liver.

### 3.5. Microarray Analysis of Liver Tissue Gene Expression Profiles

**Figure 4:**
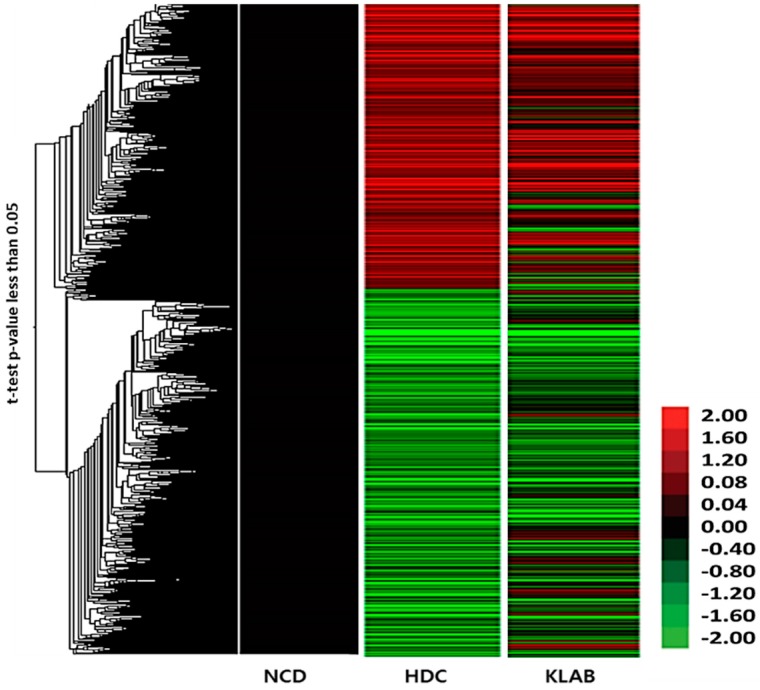
Hierarchical clustering of differentially expressed genes in the liver of *L. brevis* OPK-3 treated mice. A total of 591 deregulated genes were analyzed based on statistical significance as described in the Methods section. The heat map is represented with the expression value of each individual array.

**Figure 5:**
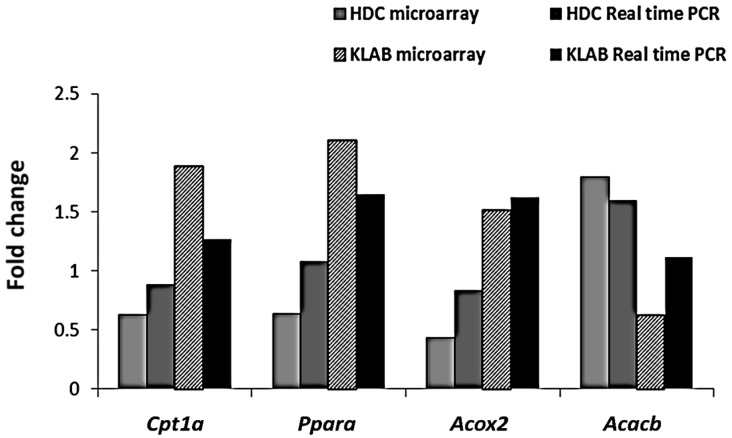
Comparison of real-time PCR (polymerase chain reaction) and microarray data analysis of *Cpt1a*, *Ppara*, *Acox2*, and *Acacb*. Gene expression patterns were consistent with microarray and real-time PCR data (*Cpt1a*, carnitine palmitoyltransferase 1a; *Ppara*, peroxisome proliferator activated receptor alpha; *Acox2*, acyl-Coenzyme A oxidase 2; *Acacb*, acetyl-Coenzyme A carboxylase beta).

We measured hepatic gene expression profiles in the HDC or KLAB group as compared with the NDC group by microarray analysis (Axon Instrument Inc., Union City, CA, USA). DNA chip analysis was done to determine differences in hepatic gene expression between the HDC and KLAB group. Treatment with KLAB caused it to be statistically significant, at least 1.5 fold up- or down-regulation of many probes set in the DNA chip. Among the 2511 genes on the oligo chip employed in this study, the expressions of 591 genes were found to be up- or down-regulated as the result of feeding high-fat diet (HDC) and were analyzed using hierarchical clustering (Figure 4). Table 3 shows selected genes differentially down- and up-regulated by KLAB based on the biology process of gene ontology descriptions. These genes, including *Gpx3*, *Ldlr*, and *Agpat5*, which are related to lipid metabolism, were overexpressed by high-fat diet, but down-regulated by KLAB supplementation. The KLAB group showed lower hepatic expression of the lipogenic gene, such as *Acacb*, than in the HDC group. In contrast, lipolytic genes such as *Ppara*, *Acox2*, and *Cpt1a* were significantly upregulated in the KLAB compared to the HDC group (Table 3). Molecules involved in response to innate immunity and inflammation, such as *Anxa1*, *Igj*, and *Myd88*, were down-regulated in the KLAB group (Table 3). The observed microarray results were confirmed using real-time PCR. Gene expression related to lipid metabolism process, including *Acacb*, *Ppara*, *Acox2*, and *Cpt1a*, were lowered after KLAB supplementation (Figure 5).

### 3.6. Expression of Adipogenic Regulating Gene in Adipose Tissue

**Figure 6:**
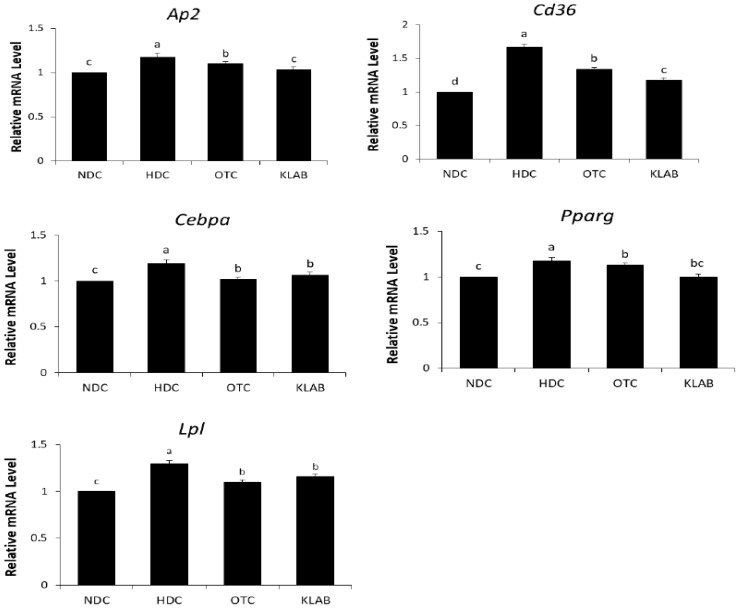
Effect of *L. brevis* OPK-3 on mRNA levels of adipogenic genes in the white adipose tissue of C57BL/6N mice. Results are Mean ± S.D of 10 mice per group. a-b-c; Values with different superscripts are significantly different by ANOVA with Duncan’s multiple range test at *p* < 0.05. NDC: Normal diet control, HDC: High fat diet control; OTC: high-fat diet plus L-ornithine 20 mg/kg; KLAB: High fat diet plus 1 × 10^9^ CFU of *L. brevis* OPK-3 per mouse. *Pparg*: Peroxisome proliferator-activated receptor gamma; *Cebpa*: CCAAT/enhancer-binding protein alpha; *Ap2*: Fatty acid binding protein; *Lpl*: Lipoprotein lipase.

The mRNA expression of adipogenesis related genes, *Pparg*, *Cebpa*, *Ap2*, *Lpl*, and *Cd36*, significantly increased in the HDC group compared to the NDC group. However, the levels of these genes in both OTC and KLAB groups were markedly lower than in the HDC group (Figure 6). Notably, mRNA levels of *Ap2* and *Cd36* were significantly decreased in the KLAB group than in the OTC group (*p* < 0.05). 

### 3.7. Expression of Inflammation Related Genes in Adipose Tissue

**Figure 7:**
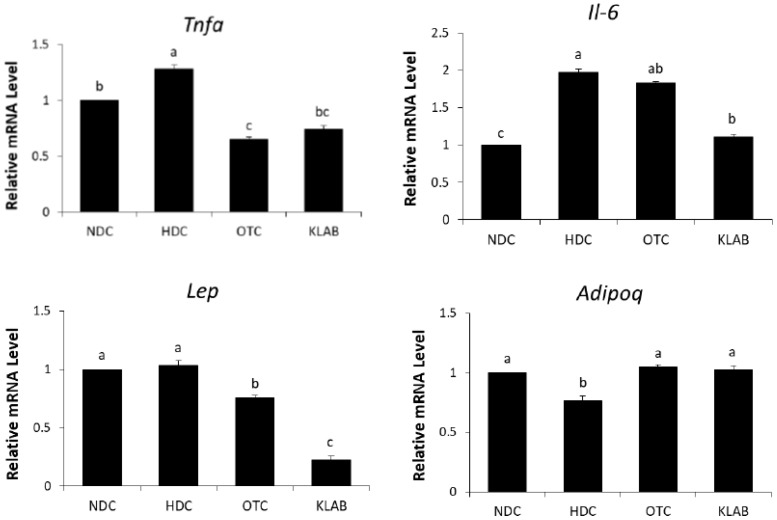
Effect of *L. brevis* OPK-3 on mRNA levels of inflammation cytokine genes in the white adipose tissue of C57BL/6N mice. Results are Mean ± S.D of 10 mice per group. a-b-c; Values with different superscripts are significantly different by ANOVA with Duncan’s multiple range test at *p* < 0.05. NDC: Normal diet control; HDC: High fat diet control; OTC: high-fat diet plus L-ornithine 20 mg/kg; KLAB: High fat diet plus 1 × 10^9^ CFU of *L. brevis* OPK-3 per mouse. *Tnfa*: Tumor necrosis factor-alpha; *Il-6*: Interleukin 6; *Lep*: Leptin; *Adipoq*: Adiponectin.

The expression levels of *Il-6*, a macrophage marker, and *Tnfa*, a pro-inflammatory cytokine, were significantly increased in the HDC group as compared with the NDC group and were down-regulated by KLAB supplementation. The relative mRNA levels of *Lep* in the KLAB were significantly lower than in the HFD group, whereas *Adipoq* mRNA was up-regulated in the OTC and KLAB groups compared to the HDC group, which was at the same range as of NDC group (Figure 7).

### 3.8. Inflammatory Cytokines in Serum

**Figure 8:**
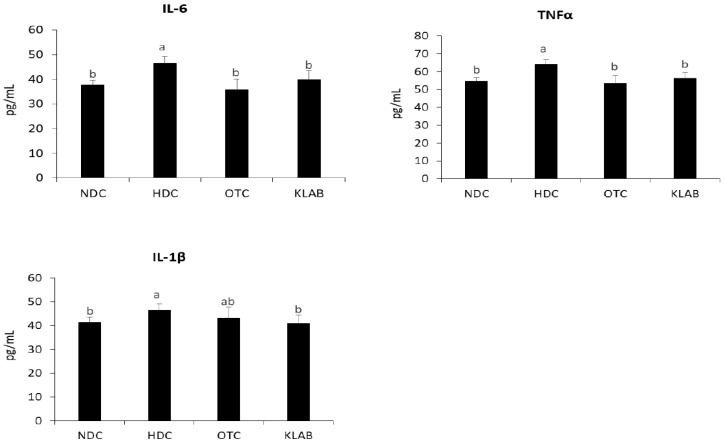
Serum levels of cytokines IL-6, IL-1β, and TNFα. a-b-c; Values with different superscripts are significantly different by ANOVA with Duncan’s multiple range test at *p* < 0.05. NDC: Normal diet control; HDC: High fat diet control; OTC: high-fat diet plus L-onithine 20 mg/kg; KLAB: High fat diet plus 1 × 10^9^ CFU of *L. brevis* OPK-3 per mouse. TNFα: Tumor necrosis factor-alpha; IL-6: Interleukin 6; IL-1β: Interleukin 1 beta.

The high-fat diet may cause inflammation status and dysregulate immune response by upregulating inflammatory cytokines, such as TNFα, IL-6, and IL-1β. We measured the concentration of serum inflammatory cytokines to determine the effect of KLAB on inflammation in obese mice. We found markedly increased concentrations of serum TNFα, IL-6, and IL-1β in the HDC group compared with those in the NDC group (*p* < 0.05). However, the KLAB supplementation prevented the rise of TNFα, IL-6, and IL-1β in serum (Figure 8). 

## 4. Discussion

Gut microbiota is changed by high-fat diet consumption but it also modulates the chronic inflammation associated with the hyperphagia and obese phenotype [33]. Some evidence suggests that several *Lactobacillus* strains have anti-obesity and anti-inflammation effects [34]. Changes of intestinal microflora and host immune response are key mechanisms of probiotic action [35]. Recent studies have suggested that the intestinal microflora is an environmental factor involved in the development of obesity-related metabolic abnormalities, supporting the possibility that probiotics may ameliorate metabolic abnormalities by modulating the gut microbiota population [36,37]. 

In this study, we demonstrated that intake of KLAB for 12 weeks prevented weight gain and increase in epididymal fat mass, and showed noticeable improvements in serum and hepatic lipid levels in the high-fat diet-induced obesity mice model. These data appear to be related to the suppression of the expression of pro-inflammatory cytokine genes in adipose tissue. We have shown that adipose tissue metabolism is changed in different ways with KLAB supplementation, and these metabolic changes are similar to changes in circulating inflammation markers. 

Probiotic supplementation is related to decreases in body weight and hepatic lipid contents [25,38]. NAFLD is caused by the synthesis of triglycerides and cholesterol in the liver due to prolonged high fat intake [39,40]. The liver is the main organ in de novo lipogenesis, which produces TG through a sequential enzymatic reaction [41]. In this study, a significant increase in the hepatic TG and TC levels was shown in the HDC group (Table 2, Figure 3). Hepatic TG levels were significantly decreased in the KLAB group, which agreed with microarray profiling and found that various genes of lipid metabolism were altered (Table 3). We then confirmed the expression of selected genes with real-time PCR. KLAB supplementation significantly lowered the expression of *Acacb*, an enzyme involved in lipogenesis, compared with the HDC group (Figure 5). In contrast, KLAB supplementation increased the mRNA levels of *Ppara*, *Acox2*, and *Cpt1a*, promoting fatty acid oxidation. These data suggest the higher lipid catabolism and lower anabolism promoting-property of KLAB (Figure 5). These results are consistent with another study that demonstrated that *Lactobacillus* prevents a decrease in *Ppara* and *cpt1a* expressions, decreases of which are implicated by fat accumulation, and improvements in other genes related to lipid metabolism such as *Fasn*, *Scd1*, and *Acaca* [33]. As a positive control, KLAB was compared with L-ornithine, which is known as a medicinal agent with an anti-obesity function through growth hormone release and basal metabolism promotion [42,43]. Consequently, the KLAB group exhibited higher or similar effects when compared to the positive control, L-ornithine. The present study demonstrated that KLAB suppressed the expression of *Adipoq* genes, such as *Pparg*, *Cebpa*, and *Ap2*, in the epididymal adipose tissues in mice. *Pparg* and *Cebpa* play vital roles in the early stage of adipose differentiation [44]. KLAB modulated the expressions of *Pparg*, *Cebpa*, *Lpl*, *Cd36*, and *Ap2* mRNA levels, which are associated with adipocyte differentiation, in the epididymal adipose tissues of mice (Figure 6). One of these genes, LPL, has no direct effect on adipocyte differentiation, but it has been reported that mRNA expression level of *Lpl* is increased by peroxisome proliferator-activated receptors (PPAR) expressed during adipocyte differentiation [45,46]. Therefore, a decrease in the expression of the *Lpl* mRNA level by KLAB supplementation (Figure 6) appears to be the result of the decreased expression levels of *Pparg* and *Tnfa* [47]. In the present study, down-regulation of *Tnfa*, *Il-6*, and *Lep*, as well as up-regulation of adiponectin, could be involved in the mechanism accounting for the decrease in epididymal adipose tissue after 12 weeks intake of KLAB (Figure 7). These results were consistent with our previous study that *L. brevis* OPK-3 was effective in down-regulating the expression of *Tnfa* and *Il-6* through inhibiting lipid accumulation in the differentiated adipocyte [27]. Therefore, we conclude that KLAB supplementation may decrease the mRNA levels of adipocyte-secreted inflammatory cytokine because KLAB has anti-adipogenic properties. In addition, KLAB supplementation increased the gene expression of adiponectin and decreased leptin expression. Adiponectin and leptin are considered to be the primary adipocytokines because they appear to be produced mainly by adipocytes. The anti-inflammatory activities of adiponectin promote the production of anti-inflammatory cytokines, IL-10 or IL-1 receptor antagonist, and inhibit pro-inflammatory cytokine IL-6 production [48,49]. Additionally, we report in the present study that concentrations of TNFα, IL-6, and IL-1β in serum were decreased by KLAB supplementation (Figure 8). TNFα, IL-6, and IL-1β levels in serum were significantly correlated with body weight, suggesting that the circulating concentrations of these cytokines may be reflected by adipose tissue mass [50]. 

Probiotics could be defined as ‘living microorganisms,’ which, when administered in appropriate amounts, provide the host of health benefits. Especially, evidence has been reported that intestinal microbiota regulates systemic energy balance through mechanisms related to the degradation and absorption of gut contents [36,51]. Obesity is associated with a specific profile of intestinal microflora, such as a decrease in the ratio of *Bacteroidetes*/*Firmicutes*, the main representative of the gut microbiota [52]. Although there are no data on factors affecting the population size and number of KLAB in colonized individuals in this study, it is assumed that the type of nutrient in the diet might have played a role in changing the population of the gut flora. Therefore, future studies are needed to investigate the compositions and metabolites of the major intestinal microflora in the animals.

## 5. Conclusions

KLAB supplementation decreases body weight and epididymal fat tissue mass in obese mice and also decreased hepatic lipid and lipogenic gene expression levels. In addition, anti-inflammatory and adipogenic genes were reduced in white adipose tissue. The results of this study suggest that improvement in the inflammatory state in the adipose tissue might be a possible underlying mechanism involved in the anti-obesity effect of KLAB without any safety or intolerance issues. Therefore, further experiments are needed to explore the anti-obesity and anti-inflammatory properties of KLAB in clinical trials.

## Figures and Tables

**Table 1:** The composition of experimental diets.

Ingredient (g)	^†^ Normal Diet	^‡^ High Fat Diet		
	NDC	HDC	OTC	KLAB
Casein, lactic	200	200	200	200
L-cystine	3	3	3	3
Corn Starch	315	-	-	-
Maltodextrin	35	125	125	125
Sucrose	350	68.8	68.8	68.8
Cellulose	50	50	50	50
Soybean Oil	25	25	25	25
Lard	20	245	245	245
Mineral Mix	10	10	10	10
Dicalcium Phosphate	13	13	13	13
Calcium Carbonate	5.5	5.5	5.5	5.5
Potassium Citrate	16.5	16.5	16.5	16.5
Vitamin Mix	10	10	10	10
Choline Bitarate	2	2	2	2
FD&C Yellow Dye #5	0.05	-	-	-
FD&C Blue Dye #1	-	0.05	0.05	0.05
Total	1055.05	773.85	773.85	773.85
Kcal	4057	4057	4057	4057
Kcal/g	3.8	5.2	5.2	5.2
Oral administration	DW	DW	L-ornithine	*Lb. brevis* OPK-3

**^†^** AIN-93 Modified diet with 4% fat (10% fat calories) content; **^‡^** AIN-93 Modified high fat diet with 35% fat (60% fat calories) content. DW: distilled water (1 mL/kg bw); NDC: Normal diet control; HDC: High fat diet control; OTC: High fat diet plus L-ornithine 20 mg/kg BW; KLAB: High fat diet plus 1 × 10^9^ CFU of *Lb. brevis* OPK-3 per mouse.

**Table 2:** The effect of *L. brevis* OPK-3 on serum and hepatic lipid profiles in mice.

Groups	NDC	HDC	OTC	KLAB
Serum (mg/dL)				
TG	94.34 ± 17.26 ^c^	140.72 ± 16.40 ^a^	109.57 ± 3.55 ^bc^	99.07 ± 11.64 ^c^
TC	155.35 ± 18.1 ^c^	314.65 ± 56.06 ^a^	304.12 ± 47.70 ^b^	307.01 ± 22.94 ^b^
HDL-c	92.89 ± 6.28 ^c^	102.57 ± 7.33 ^b^	116.32 ± 10.08 ^a^	118.50 ± 8.72 ^a^
HDL-c/TC	53.45 ± 2.22	34.07 ± 4.35	36.90 ± 2.23	36.58 ± 4.13
Liver (mg/g)				
TG	10.24 ± 2.03 ^b^	31.45 ± 4.08 ^a^	27.13 ± 2.16 ^a^	19.59 ± 5.20 ^b^
TC	0.12 ± 0.02	0.14 ± 0.02	0.12 ± 0.01	0.12 ± 0.01

Results are Mean ± S.D of 10 mice per group. a-b-c; Values with different superscripts in the same row are significantly different by ANOVA with Duncan’s multiple range test at *p* < 0.05. NDC: Normal diet control; HDC: High fat diet control; OTC: High fat diet plus L-ornithine 20 mg/kg BW; KLAB: High fat diet plus 1 × 10^9^ CFU of *L. brevis* OPK-3 per mouse.

**Table 3:** Comparison of gene expression between high-fat diet and *L. brevis* OPK-3.

Symbol	Gene Name	Fold Change		Accession ID
		HDC	KLAB	
**Immunity and inflammation response**				
*Anxa1*	annexin A1	4.01	0.61	NM_009071
*Igj*	immunoglobulin joining chain	2.67	0.54	NM_152839
*Myd88*	myeloid differentiation primary response gene 88	1.52	0.66	NM_010851
**Lipid metabolic process**				
*Slc27a1*	solute carrier family 27 (fatty acid transporter), member 1	0.58	2.15	NM_011977
*Gpx3*	glutathione peroxidase 3	3.17	0.62	NM_008161
*Ldlr*	low density lipoprotein receptor	1.50	0.65	NM_010700
*Agpat5*	1-acylglycerol-3-phosphate O-acyltransferase 5	1.74	0.64	NM_026792
*Cpt1a*	carnitine palmitoyltransferase 1a, liver	0.63	1.88	NM_013495
*Acacb*	acetyl-Coenzyme A carboxylase beta	1.80	0.62	NM_133904
*Ppara*	peroxisome proliferator activated receptor alpha	0.64	2.10	NM_011144
*Acox2*	acyl-Coenzyme A oxidase 2	0.43	1.51	NM_053115

Fold change of HDC in NDC versus HDC; Fold change of KLAB in NDC versus HDC animals. HDC: High fat diet control; KLAB: High fat diet plus 1 × 10^9^ CFU of *L. brevis* OPK-3 per mouse.
